# Nerves: Their Uses and Troubles

**Published:** 1887-07-09

**Authors:** 


### Nerves: their Uses and Troubles.

"If I had made men and women I would have taken care to make them without nerves," said a poor fellow, who was suffering from the tortures of sciatica. Possibly; and if he had made men and women they would probably not have been worth the trouble spent upon them when they were finished. Now nerves are undoubtedly troublesome and vexatious things, unless they are well managed; but so are pocket-knives to schoolboys, bicycles to youths in their teens, and lovers to young men and maidens over twenty. But what schoolboy would be without his pocket-knife, and what young man or woman would exist without a lover except on the sternest compulsion? The truth is that many of the gifts of nature have their drawbacks, and sometimes our most valued possessions are capable of giving us the greatest and most exquisite pain. This is so true that it has become a commonplace which nobody questions.

There are certain animals in existence which have no nerves, or at least none that can be traced. Are they happy and prosperous? That we don't know. What we think we do know is, that if they have no nerves they are incapable of experiencing a great many of the pleasures and delights which fall to our lot. For instance, they can't taste anything. To them a piece of red brick is as luscious as an early strawberry, and a salmon-steak would no more tickle their palate than the fishing-rod the salmon was caught with. Poor creatures—they have no palate to tickle. Of course it must be admitted that they have no pains. But if, in order to ensure freedom from pain, we are to be deprived of all our pleasures, most of us would be prepared to say, "We will risk the pain rather than lose the pleasure." Besides, it is a striking fact in nature, and one always to be remembered, that if all the world were wise, we might have all the pleasures our varying kinds of nerves are capable of giving us, with hardly any pains worth speaking of. That is a truth which should never be lost sight of in considering philosophically and practically the peculiar nature of man.

The little creatures which have no nerves are not merely destitute of the power of taste: they can neither see, nor hear, nor smell, nor think. If Mr. Mallock could publish his book in their language, and ask them if life were worth living, they might very well answer "No,"—unless they thought it might be worth living on the chance of their developing into creatures a stage or two higher, and so getting a rudimentary nervous system, with all the powers which such a system implies.

Many people seem to think that nerves were made for no other purpose than that of enabling us to feel pain. True, if they are religiously inclined they will admit that pain may have it uses. It may teach us to take care of an injured and suffering part of the body, and it may be of service as a means of discipline for the soul. Nerves do certainly serve these purposes for many people, but the range of their usefulness is infinitely wider than such a view of the case would argue. When a fly alights on the face of a sleeping child, a tickling sensation is set up, and the child is informed of the presence of something on its face. If there were no nerve-filaments in the skin of the face, a red-hot cinder might fall on the spot instead of the fly, and the child would never feel it. When the blacksmith grasps the cold end of a piece of metal which he is working, he knows by the feel of it that he is grasping the cold end. If he had no nerves in his fingers he could not tell "by the feel" whether he was grasping the cold end or the hot, not even if the skin were all roasted off. There is some use in nerves, then: they give us the power of feeling. Such nerves are called nerves of sensation, sensory nerves.

Suppose a man crossing a field gazing intently at the flowers. Suddenly he looks up, and dashes off to the nearest gate or stile like one possessed. What did the man see on the ground to make him rush off at such mad speed? Nothing! Away at the other end of the field a bull bellowed: the sound was carried by the air to the man's ear, and excited his auditory nerves. Thereupon he looked up, and the body of the bull projected an image upon the ends of his optic or eye-nerves; as the consequence of these two sensations the brain transmitted a message down the spinal cord to the nerves supplying the muscles of the legs and arms and body. These nerves of motion instantly set all the muscles in action, and away rushed the human machine to escape the horns of the bull. If the ear had had no nerves the man would not have heard the bull bellow: if he had heard it, but possessed no eye-nerves, he would not have seen it charging down upon him. If he had seen it charging down upon him, but had had no nerves in his legs, he could not have run away: and if he had not run away—why, the bull would have had some pleasant sport, but the man would not have been thankful that he was made without nerves. When a man runs, or swims, or plays the fiddle, or rides his bicycle, he does it all by means of his nerves of motion. If he had no such nerves he would be just as helpless as a log of wood.

(*To be continued.*)

---

At Bolton, during the past six months, £1,047 2*s.* 4*d.* has been received from various associations of working-men, mills, factories, workshops, etc., on behalf of the hospital. For a town of the size of Bolton, filled as it is with such a large working-class population, this sum speaks volumes for the desire of the working-men to help themselves.

